# Pharmacological ZO-1 agonist treatment attenuates ammonia nitrogen stress-induced mucosal inflammation and intestinal barrier failure in *Leiocassis longirostris*

**DOI:** 10.3389/fimmu.2026.1842403

**Published:** 2026-06-08

**Authors:** Senyue Liu, Yang Feng, Chengyan Mou, Lu Zhang, Yuanliang Duan, Zhipeng Huang, Han Zhao, Jian Zhou, Jun Du, Qiang Li, Yongqiang Deng

**Affiliations:** 1 Fisheries Research Institute, Sichuan Academy of Agricultural Sciences (Sichuan Fisheries Research Institute), Chengdu, Sichuan, China; 2 Aquatic Health and Intelligent Aquaculture Key Laboratory of Sichuan Province, Chengdu, China

**Keywords:** ECM-FA pathway, intestinal barrier, intestinal inflammation, mucosal immunity, ZO-1

## Abstract

Ammonia nitrogen is a common environmental stressor in intensive aquaculture, but the mechanisms by which it causes intestinal mucosal immune failure remain incompletely understood. Using combined *in vivo* and *ex vivo* models of *Leiocassis longirostris* along with histology, transepithelial electrical resistance (TEER) measurement, immunofluorescence, transcriptomics and qRT-PCR, we observed that ammonia exposure induced inflammatory cell infiltration in the lamina propria and disruption of the intestinal epithelial barrier. Although transcripts of core tight junction (TJ) components (such as *zo-1, occludin*) were significantly upregulated, their protein assembly at the apical membrane was severely disrupted, as indicated by fragmented TJ ultrastructure and a sharp decline in TEER. We refer to this paradoxical response as “ineffective transcriptional compensation.” ZO-1 is a key target of this transcription-function decoupling. Pharmacological stabilization of ZO-1 reduced mucosal inflammatory damage and restored TJ protein localization and barrier function without large-scale transcriptional changes. ZO-1 stabilization also partially restored the extracellular matrix-focal adhesion (ECM-FA) pathway, which contributes to mucosal integrity. The decoupling between mRNA upregulation and protein function is a characteristic feature of ammonia-induced mucosal dysregulation in this species. These results indicate that stabilizing ZO-1 may be a potential strategy to strengthen intestinal mucosal resilience in aquaculture.

## 1 Introduction

The accumulation of ammonia nitrogen (NH_3_/NH_4_^+^) in intensive aquaculture systems, primarily driven by accelerated protein metabolism and nitrogen waste, represents a critical biochemical stressor that limits production efficiency and fish health (1, 2). As a potent environmental toxicant, ammonia (particularly in its non-ionic form, NH_3_) readily diffuses across biological membranes, inducing structural and functional damage in critical organs, including the intestine (3, 4). While the fish intestine is the primary site for nutrient assimilation, it also functions as a vital mucosal immune barrier. Its functional integrity relies heavily on the biochemical stability of the epithelial tight junction (TJ) complex (5, 6). Compromise of this barrier facilitates pathogen translocation and systemic inflammation, with severe consequences for organismal health and survival (7).

The structural foundation of the intestinal physical barrier is the TJ complex between epithelial cells, composed of scaffolding proteins (such as ZO-1, ZO-2), transmembrane proteins (such as Occludin, Claudins family), and cytoskeleton-associated proteins (8, 9). It precisely regulates paracellular permeability and is the key executor of barrier function (10). Among these, ZO-1 acts as the master scaffolding protein, essential for TJ assembly and maintenance by anchoring transmembrane proteins to the actin cytoskeleton (11, 12). Furthermore, the stability of the apical TJ complex is biophysically supported by the extracellular matrix–focal adhesion (ECM-FA) system, which provides the necessary mechanical anchoring and cellular polarity for epithelial integrity (13–15). Therefore, maintaining the stability of ZO-1 and its interaction with the underlying support system is crucial for preserving the mucosal immune-physical barrier under environmental stress.

*Leiocassis longirostris (L.longirostris)* is a high-value freshwater fish endemic to China, characterized by a lack of scales and a highly permeable mucosal surface, making it an ideal model for studying the responses of the intestinal mucosal barrier to environmental stress (16). While our previous research in related species such as *Pelteobagrus fulvidraco* (*P. fulvidraco*) established a conventional response pattern where ammonia stress induces barrier failure by suppressing ZO-1 transcription (17), our preliminary observations in *L. longirostris* suggest a markedly different regulatory logic. Specifically, the intestinal barrier of this species was significantly damaged, but the related genes seemed to exhibit contradictory transcriptional upregulation under ammonia stress. Whether this response pattern represents a functional decoupling between mRNA abundance and functional protein output remains to be fully characterized.

This study aims to systematically analyze the pathological and immunological features of ammonia-induced intestinal damage in *L. longirostris* using both *in vivo* and *ex vivo* models. We specifically investigate whether the loss of barrier function is driven by an “ineffective transcriptional compensation” pattern where upregulated TJ transcripts fail to form a stable protein scaffold. Furthermore, we evaluate the potential of stabilizing ZO-1 protein homeostasis to rescue the mucosal barrier from ECM-FA dysfunction. These findings may inform the development of strategies to support intestinal barrier function in sustainable aquaculture.

## 2 Materials and methods

### 2.1 Experimental animals and grouping

A total of 180 healthy juvenile *L. longirostris* (22.18± 0.75 g; 1.56 ± 0.62 cm) were obtained from the experimental base of the Fisheries Research Institute, Sichuan Academy of Agricultural Sciences. Prior to the formal experiment, all fish were acclimated for 15 d in a recirculating aquaculture system under the following conditions: water temperature 28 ± 1 °C, dissolved oxygen (DO) concentration ≥ 6 mg L^-^¹, and pH 7.4 ± 0.2. Based on preliminary experiments, four experimental groups were established. Each group consisted of three biological replicates (independent tanks), with 15 fish per replicate (45 fish per group). The groups were designed as follows: Control group (Control, intraperitoneally injected with 20 μl physiological saline); Ammonia nitrogen stress group (AN, intraperitoneally injected with 20 μl physiological saline, then exposed to a total ammonia nitrogen (TAN) concentration of 13 mg L^-^¹ (equivalent to 1/2 24h LC_50_) in water, **Supplementary Table 1**); ZO-1 agonist group (Ago, intraperitoneally injected with 20 μL of 5 mM ZO-1 agonist (HY-N6612B, C_6_H_9_NaO_7_, MCE, China), **Supplementary Table 2**); ZO-1 agonist combined with ammonia nitrogen stress group (Ago+AN, intraperitoneally injected with 20 μL of 5 mM ZO-1 agonist, followed 24 h later by exposure to 13 mg L^-^¹ TAN). The stress exposure lasted 24 h.

### 2.2 *Ex vivo* intestinal explant culture

An *ex vivo* intestinal explant model for *L. longirostris* was established following a modified protocol described previously (18, 19). The procedure is briefly summarized as follows. Complete culture medium was prepared using Leibovitz’s L-15 medium supplemented with 15% fetal bovine serum (FBS) and 1% penicillin-streptomycin-amphotericin B. Healthy fish without prior stress exposure were anesthetized, surface-disinfected with 75% ethanol, and dissected under aseptic conditions to obtain mid-intestinal tissue. The tissue was sequentially rinsed three times with 75% ethanol, ice-cold phosphate-buffered saline (PBS), and antibiotic-containing PBS. After removing the mesentery and luminal contents, the tissue was sectioned into approximately 2-mm-thick ring-shaped explants. The explants were immediately transferred into 6-well plates (one explant per well, 5 mL culture medium per well). Each group consisted of three biological replicates, with one 6-well plate (six explants) per replicate, giving 18 explants per group in total.

Based on the preliminary experiments (**Supplementary Table 3**; **Supplementary Figure 1**), an appropriate *ex vivo* time and ammonia nitrogen concentration were selected to trigger significant but non-lethal barrier disruption. Briefly, intestinal explants were divided into four groups (Control, AN, Ago, AN+Ago) for a total culture period of 6 h. The procedure comprised two phases: a 1 h pretreatment followed by a 5 h treatment. During the pretreatment, the Control and AN groups were maintained in basal medium, whereas the Ago and AN+Ago groups were cultured in basal medium supplemented with 0.25 mM ZO-1 agonist (1/20 of the *in vivo* concentration). Subsequently, in the treatment phase, the groups received the following media for 5 h: Control (basal medium), AN (basal medium + 0.65 mg L^-^¹ TAN (1/20 of the *in vivo* concentration)), Ago (basal medium + 0.25 mM ZO-1 agonist), and AN+Ago (basal medium + 0.25 mM ZO-1 agonist + 0.65 mg L^-^¹ TAN). All explants were incubated at 25 °C with 5% CO_2_ throughout the entire 6 h culture period.

### 2.3 Sample collection

Upon completion of the *in vivo* experiments, two fish were randomly selected from each of the three replicate tanks per group, yielding six fish per group (n=6). Following anesthesia with MS-222 (100 mg L^-^¹), the fish were dissected to collect mid-intestinal tissue. Upon completion of the *ex vivo* experiments, two explants were randomly selected from each of the three replicate plates per group, yielding six explants per group (n=6). The collected samples were allocated for various analyses: histological observation (fixed in 4% paraformaldehyde), ultrastructural observation (fixed in 2.5% glutaraldehyde), RNA extraction (flash-frozen in liquid nitrogen and stored at -80 °C), and real-time measurement of transepithelial electrical resistance (TEER).

### 2.4 Histological analysis

#### 2.4.1 Hematoxylin and eosin staining

Following the method described by Liu et al. (20), tissues fixed in 4% paraformaldehyde were processed through graded dehydration, cleared in xylene, and embedded in paraffin. Sections of 5 µm thickness were obtained using a Leica RM2235 microtome (Wetzlar, Hesse, Germany). After deparaffinization in xylene, rehydration through a graded ethanol series, rinsing in distilled water, and staining, the sections were observed under a Nikon Eclipse E200 (Tokyo, Japan) to examine pathological changes in the intestinal tissue structure.

#### 2.4.2 Alcian blue-periodic acid schiff staining

Following the method described by Wang et al. (21), deparaffinized and rehydrated sections were rinsed with distilled water, oxidized with 1% periodic acid, stained with Schiff’s reagent, rinsed with sodium bisulfite solution, washed with distilled water, stained with alcian blue dye solution, washed again with distilled water, dehydrated, cleared, and mounted. The sections were then observed under a Nikon Eclipse E200 (Tokyo, Japan) to examine changes in mucous cells. ImageJ software (Version 1.8.0) was used to quantify the mucous cell density (number of cells per individual villus) and the mucous layer thickness, based on six random fields of view per group.

#### 2.4.3 Transmission electron microscopy observation

Tissues fixed in glutaraldehyde were rinsed with 0.1 M phosphate buffer and post-fixed with 1% osmium tetroxide. After dehydration through a graded acetone series, the samples were embedded in Epon 812 epoxy resin. Ultrathin sections (70 nm thickness) were prepared using a Leica UC7 ultramicrotome (Wetzlar, Hesse, Germany), double-stained with uranyl acetate and lead citrate, and then observed under a Hitachi HT7800 transmission electron microscope to capture images of intestinal microvilli and TJ ultrastructure. ImageJ software was employed to measure microvillus length and mucous granule diameter.

### 2.5 Transcriptome sequencing and data analysis

#### 2.5.1 RNA extraction, quality assessment, and sequencing

Total RNA was extracted from intestinal tissues using the Trizol method. The concentration, purity, and integrity of the RNA were subsequently assessed using a NanoDrop 2000 spectrophotometer and an Agilent 2100 Bioanalyzer. Only samples meeting the following criteria proceeded to library construction: total RNA ≥ 1 µg, concentration ≥ 45 ng µL^-^¹, A260/A280 ratio between 1.8–2.2, and A260/A230 ratio between 2.0–2.2. Prior to library construction, equal amounts of total RNA from two fish within the same replicate tank were pooled, yielding three pooled RNA samples per group. Illumina-compatible sequencing libraries were constructed from qualified mRNA. Paired-end sequencing was performed on an Illumina NovaSeq X Plus platform (Gene Denovo Biotechnology Co., Ltd., Guangzhou, China).

#### 2.5.2 Bioinformatics analysis

Raw sequencing data underwent quality control (QC) procedures, which involved the removal of reads containing adapters, low-quality reads (Q20< 20), and reads with an ‘N’ content exceeding 5%, resulting in high-quality clean data. The clean reads were aligned to the *L. longirostris* reference genome (GDR21070358-2_std_1) using HISAT2 software (version 2.2.1). The expression abundance of individual genes was quantified using RSEM software (v1.3.2) (22), generating raw read counts and FPKM (Fragments Per Kilobase of transcript per Million mapped reads) values to reflect gene expression levels. Differential expression analysis between groups was performed using the DESeq2 R package (version 1.34.0). Differentially expressed genes (DEGs) were identified based on the thresholds of |log_2_(Fold Change)| > 1 and a False Discovery Rate (FDR)< 0.05. Gene Ontology (GO) and Kyoto Encyclopedia of Genes and Genomes (KEGG) enrichment analyses were conducted on the identified DEGs.

#### 2.5.3 Gene set enrichment analysis

GSEA was performed using GSEA software (v4.2.3) with GO and KEGG databases to identify significantly enriched biological pathways. Specifically, RNA-seq data were normalized using FPKM values. Genes were ranked based on the Signal2Noise metric [(µa − µb)/(σa + σb)], where µ represents the mean expression and σ represents the standard deviation. Permutation testing (1000 permutations) was used to compute raw p-values, followed by FDR correction using the Benjamini-Hochberg method.

### 2.6 Quantitative real-time PCR validation

qRT-PCR validation was performed following the method described by Guénin et al. (23). Briefly, 1 µg of total RNA was reverse-transcribed into cDNA using the PrimeScript™ RT Reagent Kit with gDNA Eraser. Specific primers (**Supplementary Table 4**) were designed based on transcriptome sequencing results using Premier (v6.0). qRT-PCR reactions were carried out on a CFX96 Touch™ Real-Time PCR System (Bio-Rad, Hercules, CA, USA). The threshold cycle (Ct) values were normalized against *β-actin* (24), which showed stable expression across all groups in both the RNA-seq dataset and qPCR raw Ct values (P > 0.05). The relative expression levels of target genes were calculated using the 2^−ΔΔCT^ method (23).

### 2.7 Immunofluorescence staining

Paraffin-embedded sections were deparaffinized, rehydrated, and subjected to antigen retrieval using citrate buffer (pH 6.0). The sections were then permeabilized with 0.1% Triton X-100 and blocked with 3% bovine serum albumin (BSA) at room temperature for 1h. Subsequently, the sections were incubated overnight at 4 °C with a mixture of primary antibodies: rabbit anti-ZO-1 polyclonal antibody (servicebio, GB111981, 1:400) and mouse anti-Claudin-1monoclonal antibody (servicebio, GB12032, 1:400). After washing with PBS, the sections were incubated with secondary antibodies (Cy3-conjugated goat anti-rabbit IgG and Alexa Fluor 488-conjugated goat anti-mouse IgG) at room temperature for 1h in the dark. Finally, the sections were mounted with an anti-fade mounting medium containing DAPI and observed under a Nikon Eclipse Ni-U upright fluorescence microscope (Japan).

To provide semi-quantitative analysis of membrane versus cytoplasmic localization, fluorescence intensity profiles were quantified using ImageJ software (Version 1.8.0). Specifically, for each experimental group, a 5 μm line was drawn perpendicularly across the apical junctional region of the epithelial cells. The fluorescence intensity along this line was recorded using the “Plot Profile” function to generate an intensity distribution curve. The membrane-associated signal was defined as the peak intensity within a 2 μm zone centered on the point of maximal signal, while the mean intensity in the adjacent 3 μmregion was recorded as the cytoplasmic signal. The membrane-to-cytoplasmic fluorescence ratio (M/C ratio) was then calculated for each junction analyzed. To ensure statistical robustness, a minimum of 30 junctions per group (distributed across all 6 biological samples) were quantified for both ZO-1 and Claudin-1staining. A decreased M/C ratio was utilized as an indicator of protein mislocalization from the membrane to the cytoplasm.

### 2.8 Transepithelial electrical resistance measurement

TEER was measured using a voltohmmeter equipped with chopstick electrodes (EVOM2 with STX2 probe, World Precision Instruments, Florida), following a modified protocol based on Justin Scott et al. (25). Briefly, freshly isolated intestinal tissue was mounted in a perfusion chamber ensuring a tight seal at both ends. An equal volume of pre-cooled physiological saline was added to the luminal (mucosal) and abluminal (serosal) sides. The electrode tips were immersed into the solutions on each side, and the raw TEER value (R-raw) was recorded once the reading stabilized. Before each measurement, the blank resistance (R-blank) was determined using an identical measurement system containing only physiological saline without tissue. The final TEER value was calculated using the following formula:


TEER (Ω·cm2) = (R_raw − R_blank) × π × D (diameter) × L (length)

### 2.9 Enzyme activity assay

Following the manufacturer’s instructions of the respective assay kits, approximately 50 mg of intestinal tissue was homogenized on ice in pre-chilled enzyme extraction buffer at a 1:10 (w/v) ratio. The homogenate was centrifuged at 8000×g for 10 min at 4 °C. The resulting supernatant was collected, and the activities of enzymes including alanine aminotransferase (ALT, NJBI, China, C009-2-1), aspartate aminotransferase (AST, NJBI, China, C010-2-1), glutamine synthetase (GS, NJBI, China, A047-1-1), glutaminase (GLS, NJBI, China, A124-1-1) and Lactate dehydrogenase (LDH, NJBI, China, A020-2-2) were determined using a microplate reader.

### 2.10 Statistical analysis

All data are presented as mean ± standard deviation. Statistical analysis was performed using SPSSPRO. Data were first tested for normality (Shapiro-Wilk test) and homogeneity of variances (Levene’s test). If the assumptions for parametric tests were met, one-way analysis of variance (ANOVA) was applied, followed by Tukey’s HSD test for multiple comparisons between groups. Otherwise, the non-parametric Kruskal-Wallis H test was used, followed by Dunn’s test for *post-hoc* comparisons. Significance levels were defined as follows: * P< 0.05, ** P< 0.01, and *** P< 0.001. Figures were generated using GraphPad Prism (version 9.0, San Diego, CA, USA) and Adobe Illustrator CC 2023 (San Jose, CA, USA).

## 3 Result

### 3.1 Ammonia nitrogen stress induces severe mucosal injury and inflammatory infiltration, alleviated by ZO-1 agonist

**Figure 1:**
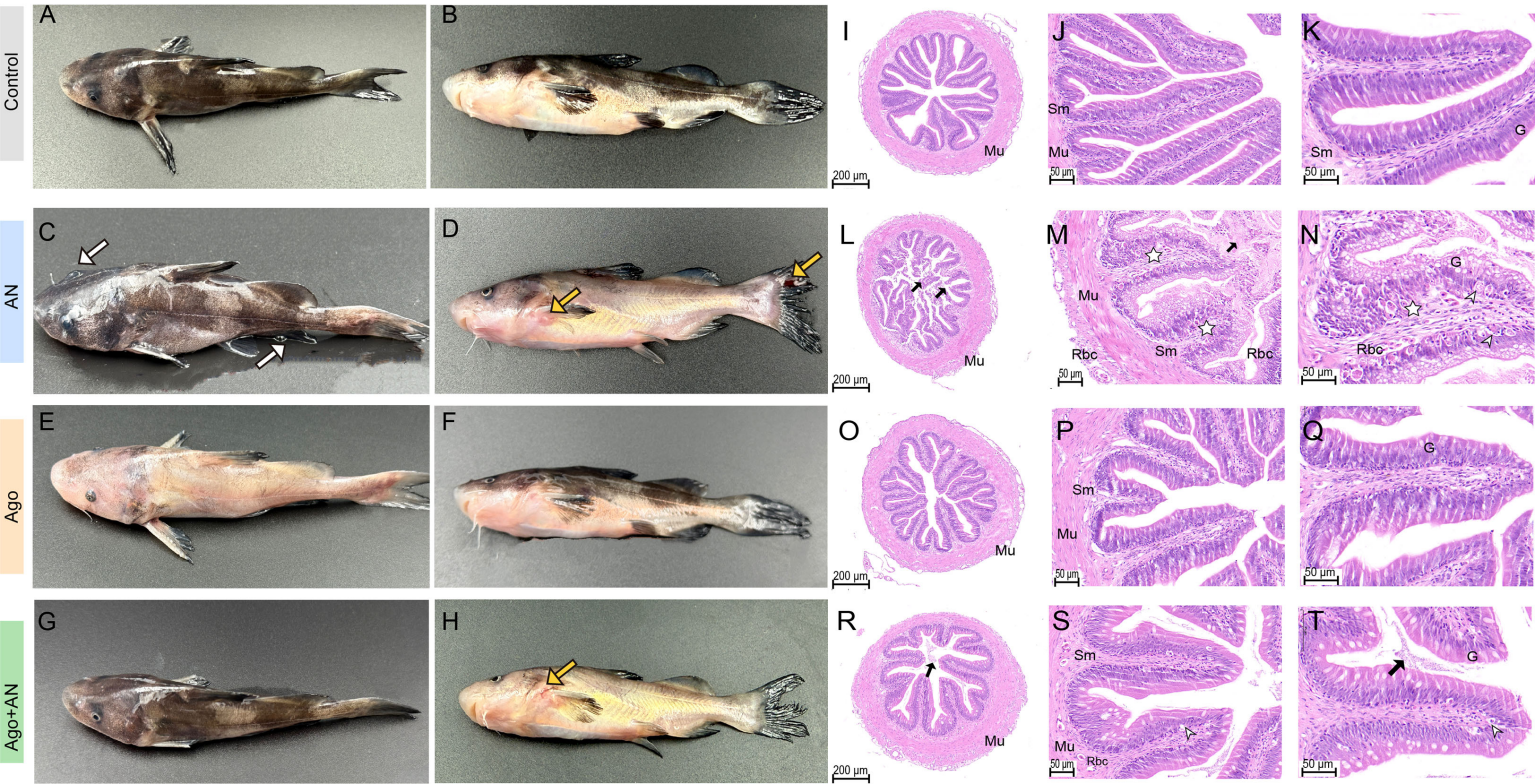
Gross morphology and H&E staining results. **(A, B)** Gross morphology of the Control group. **(C, D)** Gross morphology of the AN group, showing excessive mucus on the body surface (white arrow) and hemorrhagic spots on the pectoral and caudal fins (yellow arrow). **(E, F)** Gross morphology of the Ago group, showing no significant difference from the Control group. **(G, H)** Gross morphology of the Ago+AN group, showing significantly reduced damage compared to the AN group, with only occasional hemorrhagic spots. **(I–K)** Histological features of the Control group, showing intact mucosal structure and neatly arranged villi. **(L–N)** Histological features of the AN group, showing mucus exudation in the intestinal lumen (black arrow), accompanied by necrosis (white tailless arrow), inflammatory cell infiltration (star), and congestion. **(O–Q)** Histological features of the Ago group, similar to the Control group with no obvious pathological damage. **(R–T)** Histological features of the Ago+AN group, showing significantly alleviated intestinal mucosal damage, villus morphology tending to be regular, and occasional mucus exudation in the intestinal lumen (black arrow). Mu, mucosa; Sm, submucosa; Rbc, red blood cells; G, mucous cell.

Macroscopic observation showed smooth body surface and moderate mucus in Control group (**Figures 1A, B**). Ammonia nitrogen-stressed fish (AN group; **Figures 1C, D**) exhibited significantly increased body surface mucus and hemorrhagic spots on the pectoral and caudal fins. The ZO-1 agonist alone (Ago group) caused no significant changes (**Figures 1E, F**), while pretreatment before stress (Ago+AN group) reduced mucus overproduction and hemorrhage (**Figures 1G, H**).

H&E staining revealed intact mucosal architecture with neatly arranged villi and no inflammation in the Control group (**Figures 1I–K**). The AN group (**Figures 1L–N**) exhibited severe disruption of the intestinal mucosal immune barrier. Key pathological features included blurred epithelial boundaries, villus edema, and inflammatory cell infiltration within the lamina propria accompanied by marked congestion. The Ago group (**Figures 1O–Q**) resembled controls. Pretreatment with the agonist before stress (Ago+AN group; **Figures 1R–T**) partially alleviated damage: villus edema and breakage improved, morphology became more regular, and inflammatory cells decreased.

**Figure 2:**
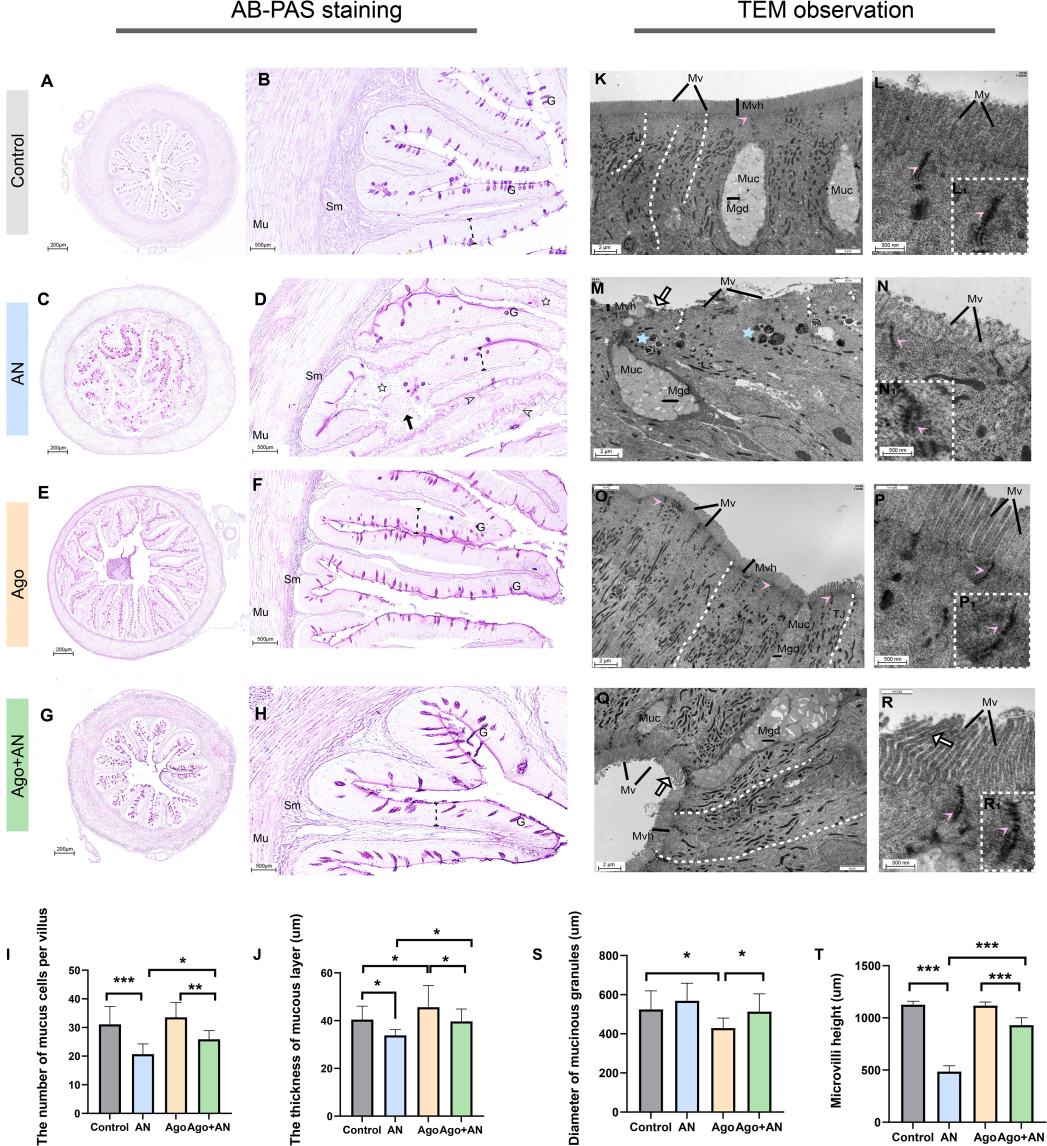
AB-PAS staining and TEM results. **(A, B)** AB-PAS staining of the Control group, showing a mucus layer of uniform thickness. **(C, D)** AB-PAS staining of the AN group. Abundant mucus is visible in the intestinal lumen (black arrow), the mucus layer thickness (black dashed line) is reduced, and some mucous cells show vacuolation (white tailless arrow) and necrosis (white star). **(E, F)** AB-PAS staining of the Ago group, showing no significant difference from the Control group. **(G, H)** AB-PAS staining of the Ago+AN group, showing restored continuity of the mucus layer compared to the AN group. **(I)** Number of mucous cells per individual villus. **(J)** Mucus layer thickness. **(K, L)** Ultrastructure of the Control group, showing neatly arranged microvilli and clear, continuous TJ structures (pink tailless arrow; white dashed line). **(M, N)** Ultrastructure of the AN group. Microvilli (white arrow) are sparse, shortened, or even shed. TJ structures (pink tailless arrow) appear blurred, loose, or fragmented, accompanied by autophagic vesicles (blue star). **(O, P)** Ultrastructure of the Ago group, similar to the Control group with no obvious pathological damage. **(Q, R)** Ultrastructure of the Ago+AN group. Damage to microvilli (white arrow) is alleviated, and TJ fragmentation (pink tailless arrow) is reduced. **(S)** Diameter of mucous granules. **(T)** Height of microvilli. *P< 0.05, ** P< 0.01, *** P< 0.001. Mu, mucosa; Sm, submucosa; G, goblet cells; Mv, microvilli; Mvh, microvillus height; Muc, mucous granules; Mgd, mucous granule diameter.

AB-PAS staining showed a continuous, uniform mucus layer and a moderate numbers of mucous cells in Control group (**Figures 2A, B**). In contrast, the AN group (**Figures 2C, D**) displayed a severely compromised mucus barrier: the layer was fragmented and partially shed, with mucous cell vacuolation and necrosis, and significantly reduced cell density (**Figure 2I**) and layer thickness (**Figure 2J**). The distribution and morphology of mucous cell, as well as the status of the mucus layer in the Ago group (**Figures 2E, F**), were similar to those in the Control group. In the Ago+AN group (**Figures 2G, H**), the continuity of the mucus layer was restored compared to the AN group. Both mucous cell density (**Figure 2I**) and mucus layer thickness (**Figure 2J**) showed recovery.

TEM further revealed ultrastructural damage. In the Control group (**Figures 2K, L**), the apical surface of intestinal epithelial cells was covered by dense and regularly arranged microvilli. The TJ structures between cells were clear and exhibited a characteristic “zipper-like” array. In the AN group (**Figures 2M, N**), stress caused sparse, shortened microvilli and blurred, loose, or fragmented TJs, with significantly reduced mucous granule diameter (**Figure 2S**) and microvillus height (**Figure 2T**). The ultrastructure in the Ago group (**Figures 2O, P**) was consistent with the Control group, showing no obvious damage. In the Ago+AN group (**Figures 2Q, R**), the damage to microvilli was mitigated, with a more orderly arrangement. TJ fragmentation was reduced, and the continuity of TJ, the diameter of mucous granules (**Figure 2S**), and the height of microvilli (**Figure 2T**) were significantly improved.

These results indicate that ammonia nitrogen stress is associated with damage to key structures of the intestinal physical barrier, and that pretreatment with a ZO-1 agonist is associated with attenuation of these changes.

### 3.2 Transcriptomic overview of the response to ammonia stress and ZO-1 agonist pretreatment

**Figure 3:**
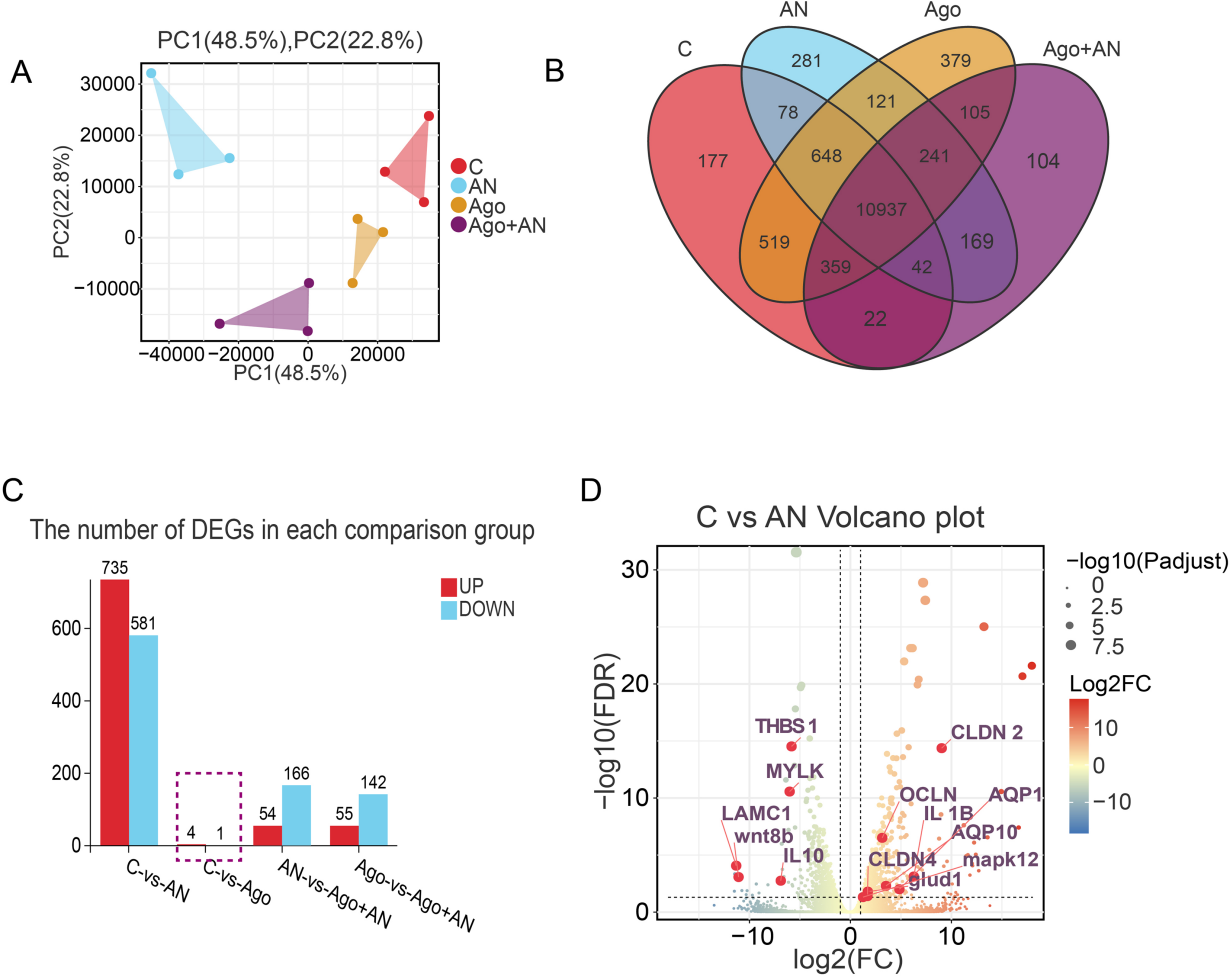
Transcriptomic analysis and DEG profiling. **(A, B)** Relationship analysis of transcriptomic data. **(A)** Principal component analysis (PCA) of four groups. **(B)** The Venn diagram shows common genes and specific genes between four groups. **(C)** Statistics of DEGs. Bar plot showing the number of DEGs (filtered with thresholds of |log_2_(Fold Change)| > 1 and FDR< 0.05) for the indicated pairwise comparisons. Note the negligible number of DEGs between the Control and Ago groups. **(D)** Volcano plots of DEGs between Control vs. AN. Orange dots represent up-regulated DEGs, blue dots represent down-regulated DEGs, and yellow dots represent non-differentially expressed genes. Selected key gene names are labeled.

Principal component analysis (PCA; **Figure 3A**) indicated that the expression profile of the AN group was distinctly separated from the Control group, whereas ZO-1 agonist pretreatment promoted a partial reversion toward the steady state. A Venn diagram (**Figure 3B**) identified a large number of shared and unique genes among the groups.

Differential expression analysis (**Figure 3C**) revealed a total of 1,316 significant DEGs in the Control vs. AN comparison, including 732 upregulated and 584 downregulated genes. The core volcano plot (**Figure 3D**) highlights the functional characteristics of these DEGs: ammonia exposure induced upregulated TJ-related genes, nitrogen metabolism-related genes, and downregulated ECM-FA pathway genes.

Notably, in the Control vs. Ago comparison, only 5 significant DEGs were identified (**Figure 3C**), all of which showed low-abundance fluctuations. This result suggests that the agonist has a minimal impact on the global intestinal transcriptional homeostasis under normal physiological conditions, and its subsequent protective effects likely involve mechanisms beyond transcriptional regulation. Detailed GO enrichment, KEGG pathway analyses, and secondary comparison volcano plots are provided in **Supplementary Figures 2**, **S3**.

### 3.3 Ammonia nitrogen stress induces intestinal nitrogen metabolism disorder

**Figure 4:**
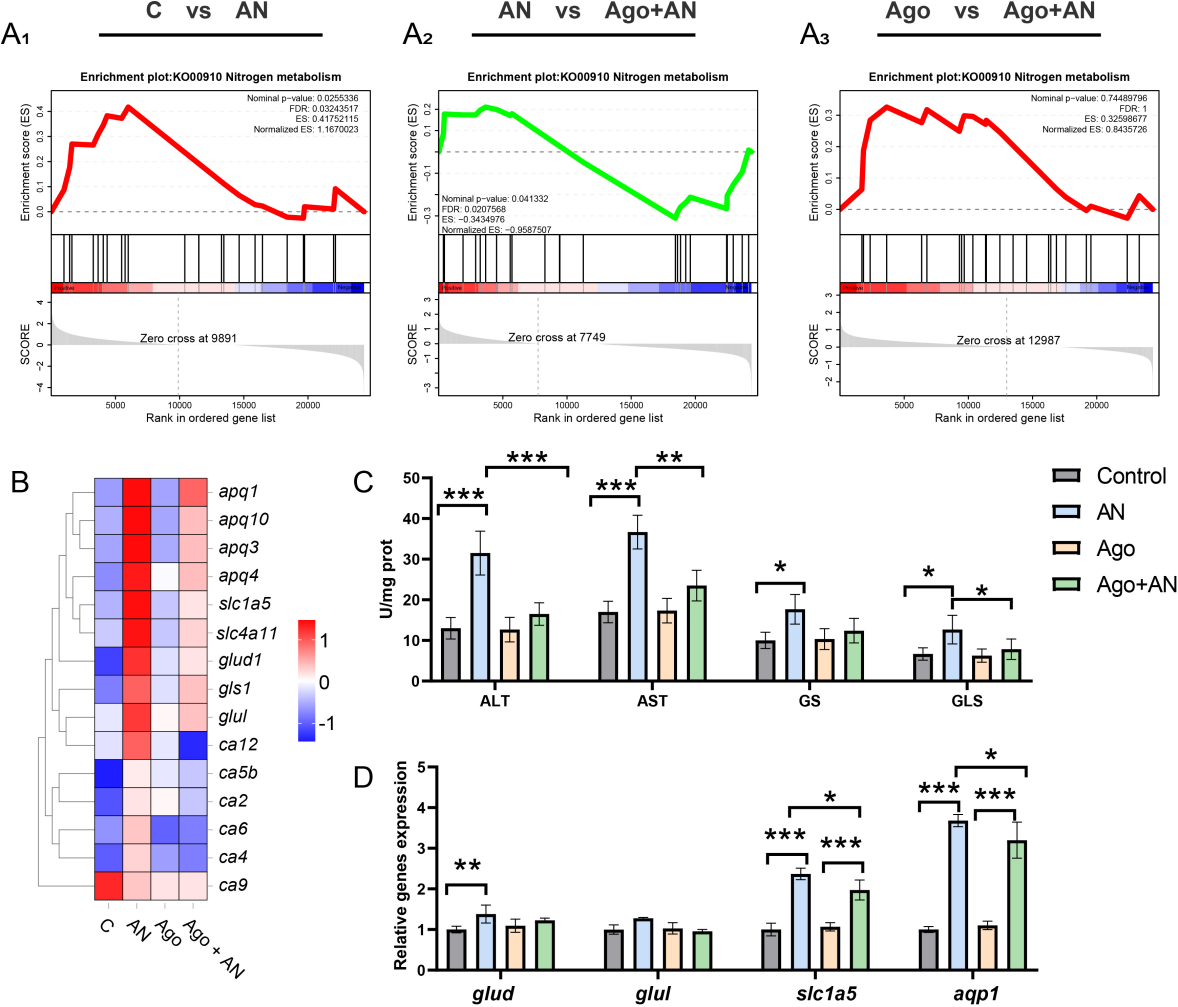
Impact of ammonia nitrogen stress on nitrogen metabolism and the mitigating effect of ZO-1 agonist. **(A1–A3)** GSEA of the nitrogen metabolism pathway for the comparisons Control vs. AN, AN vs. Ago+AN, and Ago vs. Ago+AN, respectively. **(B)** Hierarchical clustering analysis of key genes involved in the nitrogen metabolism pathway. **(C)** Enzyme activity assays related to nitrogen metabolism. **(D)** Relative mRNA expression levels of key genes related to nitrogen metabolism. Different numbers of * indicate significance: * P < 0.05, ** P < 0.01, *** P < 0.001.

GSEA confirmed significant enrichment of the nitrogen metabolism pathway (**Figure 4A**). A heatmap (**Figure 4B**) of pathway genes revealed that, compared to the Control group, the expression of genes associated with ammonia detoxification and ammonia/ion transport was broadly upregulated in the AN group. No significant changes in the expression of these genes were observed between the Control and Ago groups. In the Ago+AN group, the expression levels of these genes were significantly downregulated compared to the AN group but remained higher than in the Ago group.

Consistently, activities of GS, GLS, ALT, and AST were elevated in the AN group; in the Ago+AN group, these activities were lower than in the AN group (**Figure 4C**). qRT-PCR validation (**Figure 4D**) yielded results consistent with the enzyme activity data. In the AN group, key genes involved in nitrogen metabolism, including the ammonia-producing gene *glud*, the ammonia detoxification gene *glul*, the ion transporter gene *slc1a5*, and the aquaporin gene *aqp1*, were upregulated compared to the Control group, consistent with ammonia-induced nitrogen metabolism disorder. In the Ago+AN group compared to the AN group, the expression of *slc1a5* and *aqp1* was significantly normalized. Although the changes in *glud1* and *glul* expression were not statistically significant but showed a decreasing trend, suggesting partial amelioration of the nitrogen metabolism disorder by the ZO-1 agonist.

### 3.4 The ZO-1 agonist is associated with preservation of TJ protein complex organization

**Figure 5:**
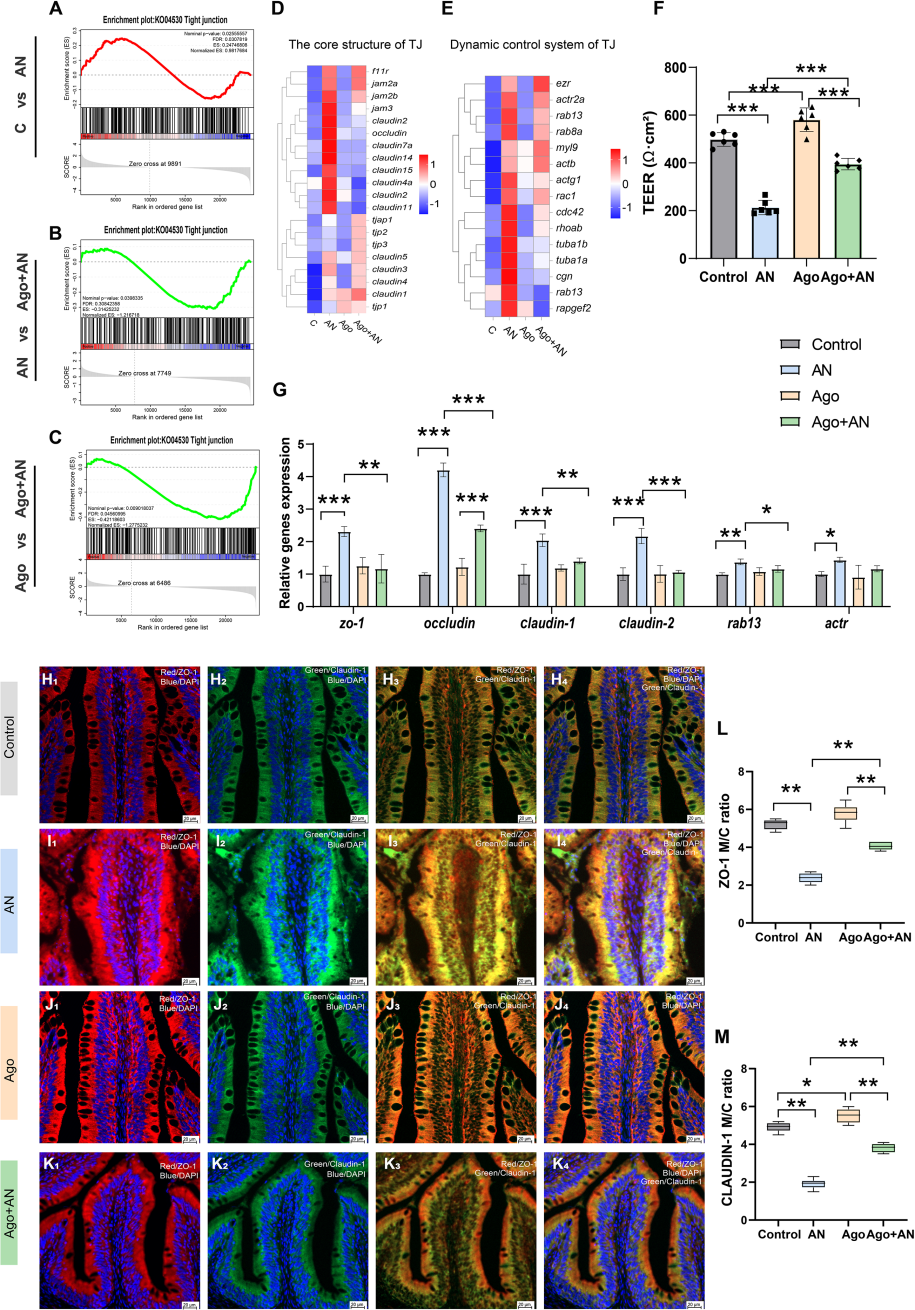
Ammonia nitrogen stress induces ineffective compensatory upregulation of TJ core components and dynamic regulators, and the mitigating effect of ZO-1 agonis. **(A–C)** GSEA of the TJ pathway for the comparisons Control vs. AN, AN vs. Ago+AN, and Ago vs. Ago+AN, respectively. **(D, E)** Hierarchical clustering analysis of key genes involved in **(D)** the core structure of TJs and **(E)** the dynamic control system of TJs. **(F)** Measurement of TEER.**(G)** Relative mRNA expression levels of key genes involved in TJ regulation. (**H–K)** Representative immunofluorescence staining images from the **(H)** Control, **(I)** AN, **(J)** Ago, and **(K)** Ago+AN groups. **(L, M)** Quantification of M/C ratio for ZO-1 and Claudin-1. Red: ZO-1; Green: Claudin-1; Blue: DAPI (nuclei). *P< 0.05, ** P< 0.01, *** P< 0.001.

GSEA (**Figures 5A–C**) revealed that the TJ pathway (KO04530) was significantly upregulated in the Control vs. AN comparison, downregulated in the AN vs. Ago+AN comparison, and the Ago vs. Ago+AN comparison. Heatmap (**Figures 5D, E**) and qRT-PCR (**Figure 5G**) analyses showed that, compared to the Control group, the mRNA levels of core TJ genes were strongly upregulated in the AN group, including barrier-forming genes (*zo-1, occludin, claudin-1*) and the pore-forming gene *claudin-2*. Concurrently, genes involved in TJ dynamic assembly, membrane trafficking, and cytoskeletal regulation (such as *rab13, actr2*) were also significantly upregulated. No significant changes were observed between the Control and Ago groups. And, compared to the AN group, treatment with the ZO-1 agonist partially normalized these abnormally high expression levels.

Despite the upregulation of TJ-related transcripts, TEER decreased significantly in the AN group compared to the Control group (**Figure 5F**), consistent with increased barrier permeability. TEER in the Ago+AN group was significantly higher than in the AN group, suggesting partial restoration of barrier function by ZO-1 agonist pretreatment.

Furthermore, immunofluorescence analysis (**Figures 5H–K**) revealed distinct patterns: In the Control group (**Figure 5H**), ZO-1 (red) and Claudin-1(green) exhibited continuous, sharp linear distribution along the apical membrane of intestinal epithelial cells, with good co-localization, indicating intact TJ structure. In the AN group (**Figure 5I**), the localization of ZO-1 and Claudin-1proteins at the apical membrane was severely disrupted. The continuous linear distribution was lost, signals became diffuse and fragmented, and semi-quantitative analysis confirmed a significant decrease in the M/C ratio for both proteins (**Figures 5L, M**), consistent with protein mislocalization. The Ago group (**Figure 5J**) showed a pattern similar to the Control group, with no significant change in the M/C ratio. In the Ago+AN group, membrane localization was partially restored (**Figure 5K**), accompanied by a corresponding increase in the M/C ratio.

Taken together, these findings reveal a paradoxical stress response in which TJ transcripts are strongly upregulated yet their protein products fail to assemble and localize correctly at the apical membrane, leading to barrier failure. We refer to this phenomenon as “ineffective transcriptional compensation”.

### 3.5 Ammonia nitrogen stress is associated with ECM-FA pathway suppression, partially reversed by ZO-1 agonist pretreatment

**Figure 6:**
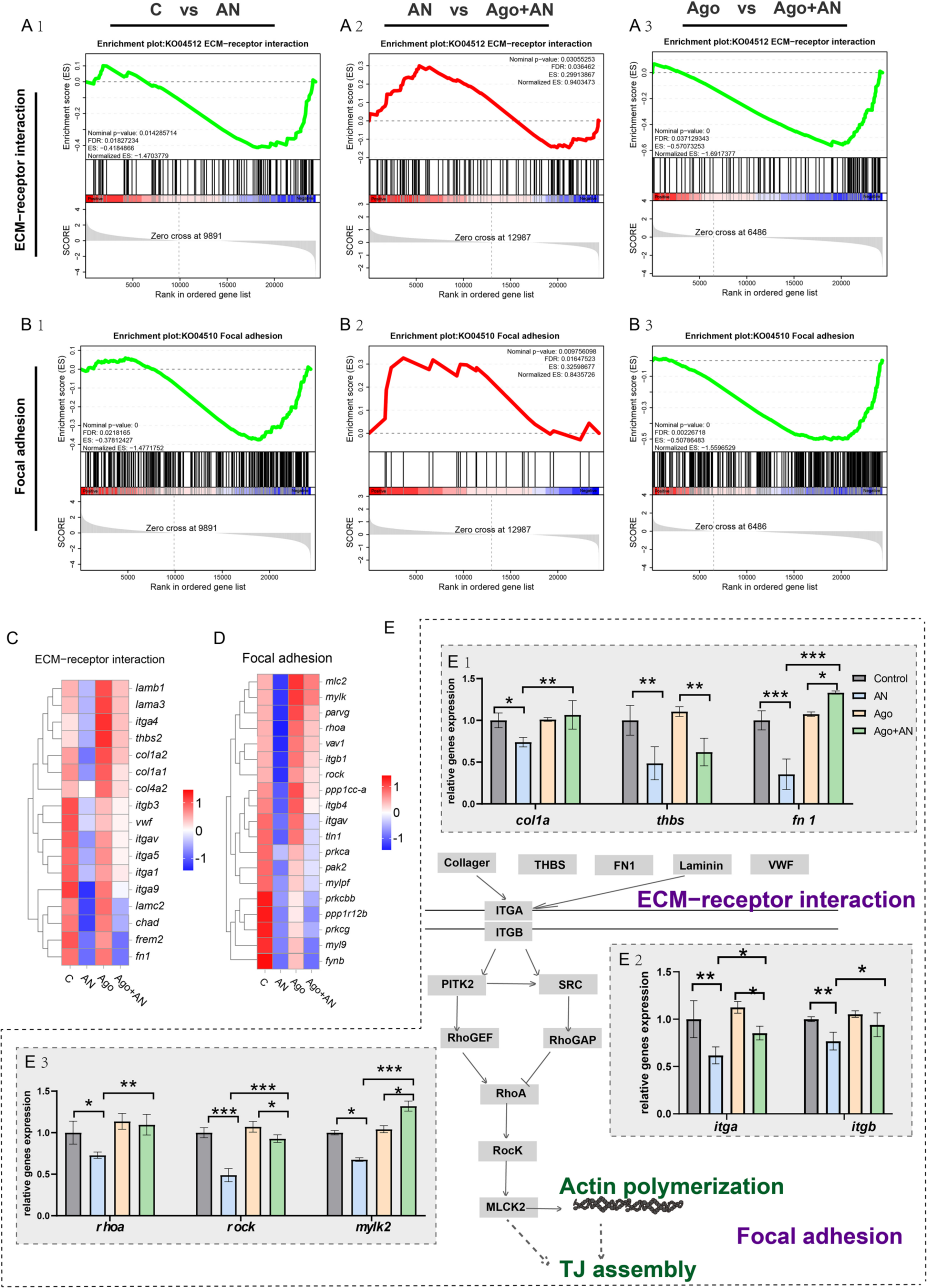
Impact of ammonia nitrogen stress on the intestinal ecm-fa pathway and the mitigating effect of the ZO-1 agonist. **(A1–A3)** GSEA of the ECM-receptor interaction pathway for the comparisons Control vs. AN, AN vs. Ago+AN, and Ago vs. Ago+AN, respectively. **(B1–B3)** GSEA of the focal adhesion pathway for the comparisons Control vs. AN, AN vs. Ago+AN, and Ago vs. Ago+AN, respectively. **(C, D)** Hierarchical clustering analysis of key genes involved in the **(C)** ECM-receptor interaction and **(D)** focal adhesion pathways. **(E)** Schematic diagram illustrating the functional linkage between the ECM-FA complex and TJs. The ECM-FA complex provides basal and lateral anchoring and mechanical support for epithelial cells, forming the foundation for maintaining cellular polarity and the stability of apical TJs. **(E1–E3)** Relative mRNA expression levels of core genes in the (E1) ECM pathway, (E2) ECM-FA interaction, and (E3) FA pathway. *P< 0.05, ** P< 0.01, *** P< 0.001.

GSEA revealed that both the ECM-receptor interaction pathway (KO04512; **Figure 6A**) and the focal adhesion pathway (KO04510; **Figure 6B**) were significantly downregulated in the Control vs. AN comparison, upregulated in the AN vs. Ago+AN comparison, and downregulated in the Ago vs. Ago+AN comparison. Heatmap analysis showed that, compared to the Control group, transcriptional expression of core ECM-receptor regulators (**Figure 6C**) and focal adhesion core regulators (**Figure 6D**) was generally downregulated in the AN group. No significant changes in these gene transcripts were observed between the Control and Ago groups. In the Ago+AN group, the expression levels of these transcripts were significantly upregulated compared to the AN group but remained lower than in the Ago group.

qRT-PCR validation (**Figure 6E**) confirmed these observations. Compared to the Control group, the AN group showed significant downregulation in the expression of ECM pathway genes (*col1a1*, *thbs*, *fn1*), ECM-FA interaction genes (*itga*, *itgb*), and FA pathway genes (*rhoa*, *rock*, *mylk2*). These findings suggest that ammonia nitrogen stress is associated with disruption of the structural support provided by the ECM-FA system. ZO-1 agonist pretreatment partially restored the expression of these genes.

The ECM-FA complex provides basal and lateral anchoring and mechanical support to epithelial cells, forming the foundation for apical TJ stability (**Figure 6E**). Therefore, these results suggest that ammonia stress weakens the basal epithelial support structure, which may contribute to TJ instability. Pretreatment with the ZO-1 agonist was associated with partial alleviation of this effect.

### 3.6 *Ex vivo* intestinal explant model validates core findings

To validate the core mechanisms in a controlled setting excluding systemic influences, we established an *ex vivo* mid-intestinal explant model. The results were consistent with the *in vivo* observations.

**Figure 7:**
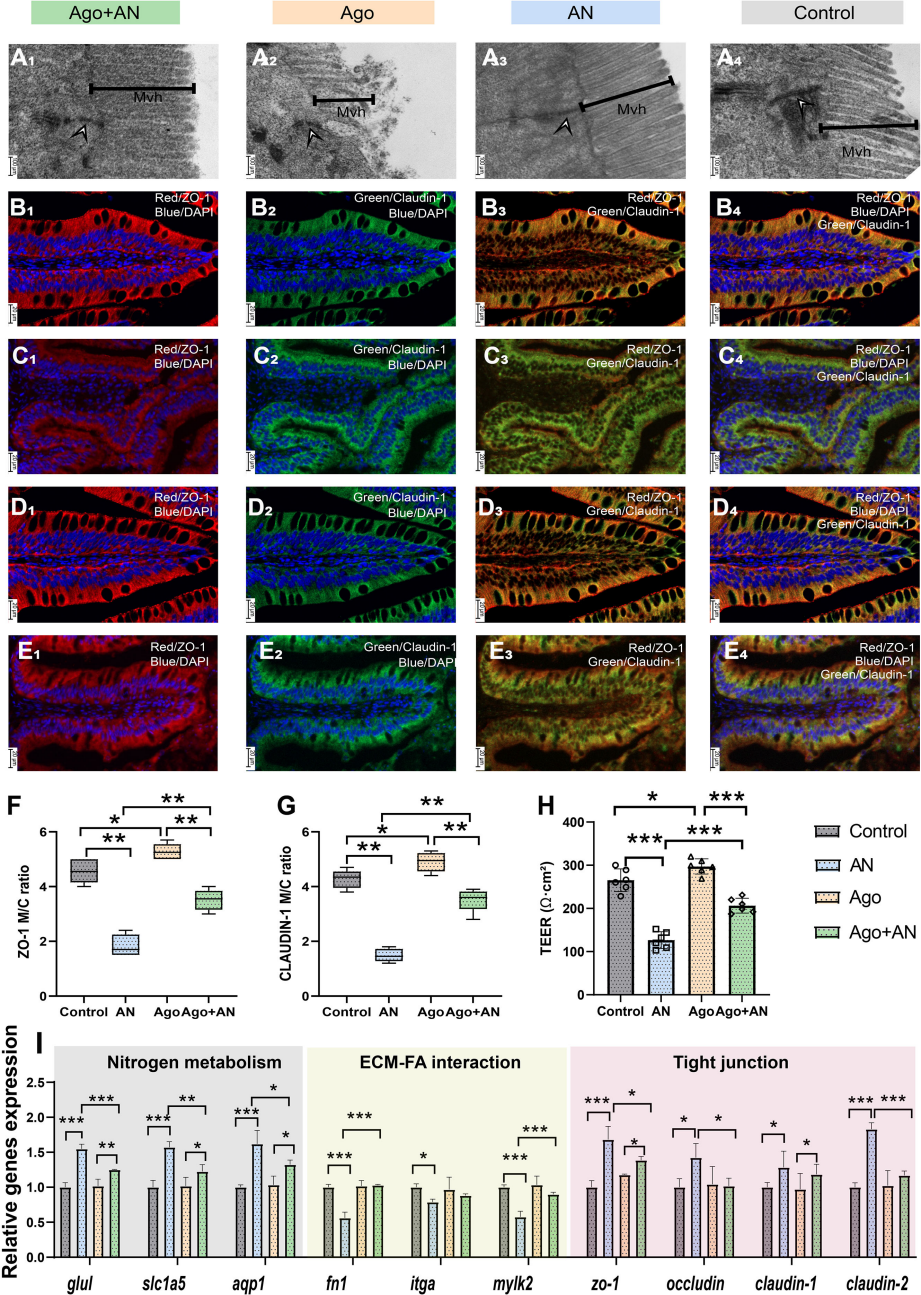
Validation using *ex vivo* intestinal explant model. **(A1–A4)** Ultrastructure of explants under *ex vivo* conditions from the (A1) Control, (A2) AN, (A3) Ago, and (A4) Ago+AN groups. Ammonia nitrogen stress resulted in sparse and shortened microvilli, as well as blurred and fragmented TJs (white tailless arrow). Treatment with the ZO-1 agonist alleviated these damages to a certain extent. **(B–E)** Representative immunofluorescence staining images of explants under *ex vivo* conditions from the **(B)** Control, **(C)** AN, **(D)** Ago, and **(E)** Ago+AN groups. Red: ZO-1; Green: Claudin-1; Blue: DAPI (nuclei). **(F–G)** Quantification of M/C ratio for ZO-1 and Claudin-1. **(H)** Measurement of TEER under *ex vivo* conditions. **(I)** Relative mRNA expression levels of ammonia metabolism-related genes, ECM-FA-related genes, and TJ-related genes under *ex vivo* conditions. Mvh, microvillus height. *P< 0.05, **P< 0.01, ***P< 0.001.

TEM analysis revealed that, compared to the Control explants (**Figure 7A1**), those exposed to ammonia nitrogen stress (AN group; **Figure 7A2**) exhibited clear ultrastructural damage, including fragmentation of TJs and significant shortening of microvilli. Explants treated with the ZO-1 agonist alone (Ago group; **Figure 7A3**) showed well-preserved or even enhanced TJ integrity. Notably, pretreatment with the agonist prior to ammonia exposure (Ago+AN group; **Figure 7A4**) partially alleviated the stress-induced damage to both TJs and microvilli.

Immunofluorescence staining provided further confirmation. In Control explants (**Figure 7B**), ZO-1 (red) and Claudin-1(green) displayed strong, continuous linear signals along cell borders, indicating intact TJ structure. The AN group (**Figure 7C**) exhibited weakened, disorganized, and diffusely distributed fluorescence signals, with a decreased M/C ratio for both ZO-1 and Claudin-1 (**Figures 7F, G**), consistent with compromised TJ structure. The Ago group (**Figure 7D**) exhibited intense and continuous signals. In the Ago+AN group (**Figure 7E**), the continuity of the signals was partially restored, with a corresponding increase in the M/C ratio compared to the AN group (**Figures 7F, G**).

Functional assessment by TEER measurement (**Figure 7H**) showed a significant decrease in the AN group compared to the Control after 6h of culture, consistent with increased paracellular permeability. Treatment with the ZO-1 agonist alone significantly increased TEER relative to the Control. Importantly, in the AN+Ago group, TEER was significantly elevated from the low point observed in the stress group, indicating a partial restoration of barrier resistance.

At the molecular level, qRT-PCR analysis (**Figure 7I**) of the explants reproduced the gene expression pattern observed *in vivo*. Ammonia stress upregulated ammonia metabolism-related genes and TJ genes, and downregulated ECM-FA-related genes, consistent with the *in vivo* results. The Ago+AN group showed significant normalization of these genes.

Collectively, the preserved ultrastructure, stable TEER, and intact TJ protein localization observed in the Control explants after the 6 h-culture period confirm the viability and suitability of this *ex vivo* model for short-term mechanistic studies. These findings are consistent with the *in vivo* observations, suggesting that ammonia exposure impairs the TJ barrier at the tissue level and that ZO-1 agonist pretreatment is associated with protection against this impairment.

### 3.7 Proposed mechanism for ZO-1-mediated intestinal TJ barrier injury under ammonia stress

**Figure 8:**
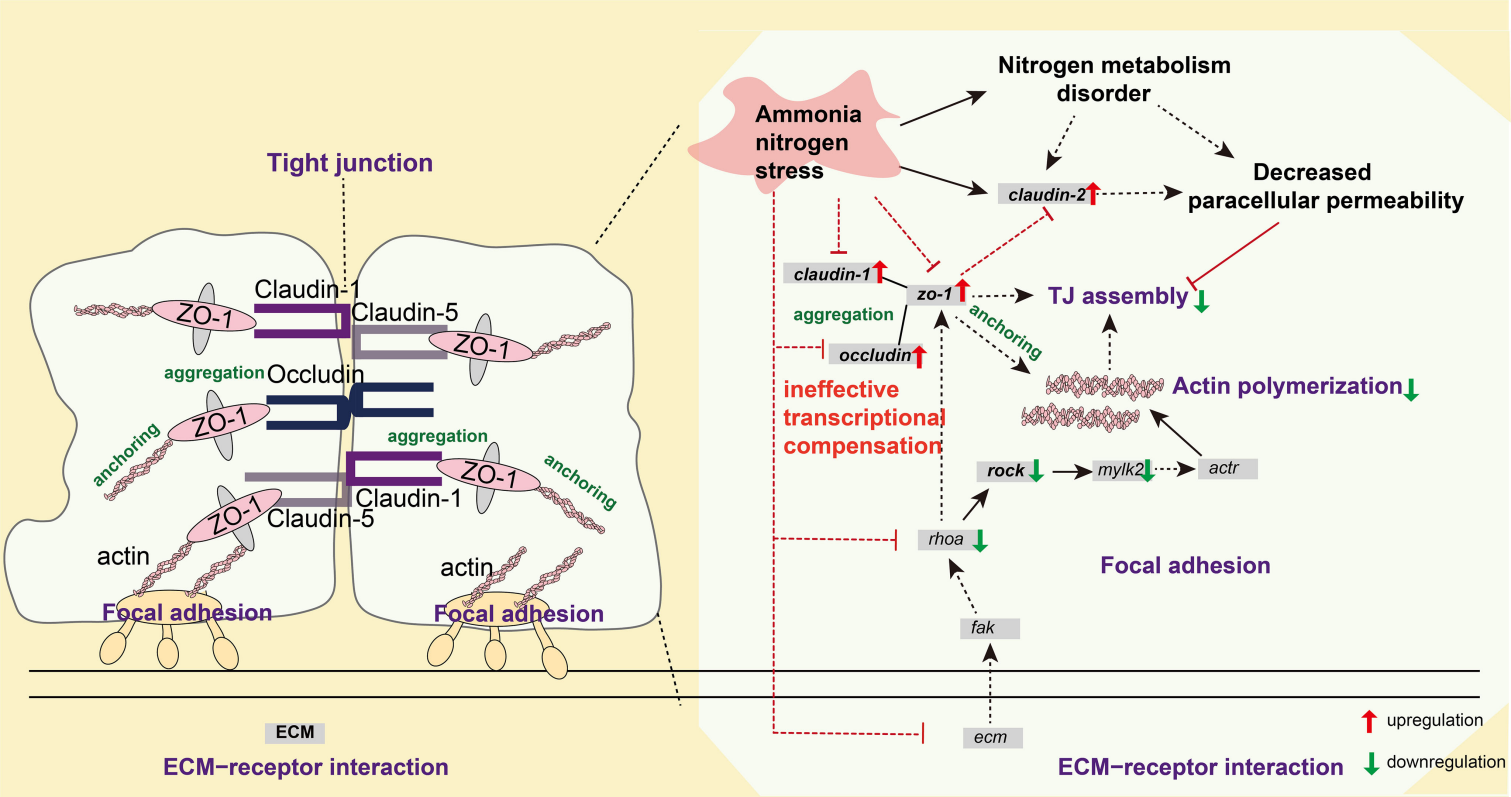
Possible mode of action by which ZO-1-associated junctional organization maintains intestinal barrier integrity under ammonia nitrogen stress. Solid arrows indicate direct regulatory pathways, and dashed arrows indicate indirect regulatory pathways.

Based on the collective findings, we propose a working model (**Figure 8**). In this model, ammonia nitrogen stress is associated with increased intracellular ammonia load and nitrogen metabolism disorder. This is accompanied by a paradoxical transcriptional response: compensatory upregulation of TJ component genes alongside downregulation of ECM-FA support genes. Under these conditions, despite the transcriptional upregulation, TJ proteins fail to assemble and anchor correctly at the apical membrane, and TJ ultrastructural disruption and barrier loss are observed. The protective effect of the ZO-1 agonist is consistent with improved preservation of ZO-1-associated junctional organization, which may facilitate barrier restoration without requiring transcriptional reprogramming.

## 4 Discussion

This study established both *in vivo* and *ex vivo* models of ammonia nitrogen stress in *L. longirostris* to investigate ammonia-induced intestinal barrier injury and the protective role of the scaffolding protein ZO-1. We describe an “ineffective transcriptional compensation” response pattern, and our data suggest that preservation of ZO-1-associated junctional structure is associated with partial restoration of barrier function.

### 4.1 Characteristics of intestinal barrier damage and the phenomenon of “ineffective transcriptional compensation”

In stress biology, environmental challenges often trigger a biochemical “decoupling” between mRNA abundance and functional protein output (26, 27). This study reports a striking manifestation of this decoupling in the *L. longirostris* intestine under ammonia stress: a broad compensatory upregulation of genes encoding core TJ components occurs alongside their failure to assemble and localize correctly, resulting in loss of barrier function. We refer to this paradoxical combination of transcriptional upregulation and functional loss as “ineffective transcriptional compensation,” and consider it a characteristic manifestation of molecular dysregulation during ammonia nitrogen toxicity.

Consistent with reports on various environmental stressors (28, 29), ammonia nitrogen stress induced classic signs of barrier dysfunction, including histopathological damage, disrupted TJ ultrastructure, and a significant decrease in TEER. However, unlike common models where TJ-genes expression is typically downregulated, our transcriptomic data revealed a critical contradiction: alongside severe TJ structural damage, the expression of genes encoding core TJ components (*zo-1*, *occludin*, *claudin-1*, *claudin-2*), and dynamic regulatory factors (*rab13*, *actr2*) shows extensive upregulation. This phenomenon suggests that under ammonia stress, the mucosal system initiates a transcriptional repair program, but the resulting transcripts do not lead to the formation of functional protein complexes, potentially increasing the metabolic burden on the cells.

### 4.2 ZO-1 agonist pretreatment is associated with partial restoration of mucosal barrier function without transcriptional reprogramming

This study examined the protective effects of the ZO-1 agonist. Briefly, under ammonia nitrogen stress, while TJ-related genes were upregulated, functional assessment revealed a stark paradox: TEER decreased substantially, TJ ultrastructure disintegrated, and membrane protein localization was disrupted. This divergence between transcription and function suggests that transcriptional activation alone is insufficient to produce a functional TJ complex under these conditions. This disruption may result from abnormal post-translational modifications (PTMs) of TJ proteins (30) or the depletion of molecular chaperones essential for protein folding and assembly under ammonia-induced stress (31).

ZO-1 agonist pretreatment was associated with partial alleviation of the aforementioned damage. This effect does not appear to involve transcriptional reprogramming, but rather is associated with normalization of stress-induced aberrant expression and improved TJ protein assembly at the membrane. Transcriptomic data further support this conclusion: only five low-abundance DEGs were identified between the Ago group and the Control group, with no pathway-level transcriptional reprogramming. These observations are consistent with a protein-level mode of action that does not depend on transcriptional reprogramming.

As the core TJ scaffolding protein, ZO-1 links transmembrane proteins (Occludin, Claudins) (11, 12) to the cytoplasmic actin cytoskeleton (32). ZO-1 agonists may exert protective effects through improved preservation of ZO-1-associated junctional structure. This protection appears to involve multiple coordinated effects: it is associated with repair of the ECM-FA structural support system, which may reduce mechanical stress on TJs and provide a more stable foundation for barrier integrity (33); and it partially alleviates nitrogen metabolism disorder, which may reduce intracellular ammonia accumulation and create a more favorable microenvironment for barrier repair.

### 4.3 Disruption of the ECM-FA pathway exacerbates TJ instability

This study reports that ammonia nitrogen stress is associated with downregulation of the intestinal ECM-FA pathway in *L. longirostris*, adding to the understanding of barrier injury in aquatic animals. Ammonia nitrogen stress significantly downregulated core ECM-FA genes, impairing the basal and lateral mechanical support for the intestinal epithelium. The ECM-FA complex serves as the mechanical anchoring foundation for epithelial cells, not only providing a stable mechanical environment (34) but also acting as a crucial structural support for maintaining cell polarity and the stability of apical TJs (33, 35). Its dysfunction may increase the mechanical stress on the apical TJ complex, further aggravating TJ structural disintegration (36).

It is noteworthy that the weakening of this basal support structure also alters the mechanical environment experienced by the apical TJ complex, leading to a further decline in its stability. These observations suggest a dual contribution to barrier disruption: direct TJ damage and weakened basal structural support. Interestingly, ZO-1 agonist treatment was associated with partial restoration of ECM-FA-related gene expression. This raises the possibility that improved preservation of TJ complex structure may indirectly support the maintenance of basal adhesion structures, although the underlying mechanisms remain to be investigated.

### 4.4 Role of nitrogen metabolism disorder in barrier damage

Ammonia stress activated local intestinal nitrogen metabolism pathways, evidenced by the upregulation of detoxification (*glul*) and ammonia-generating/transport genes (*glud1*, *aqp1*, *slc1a5*) and increased enzyme activities (GS, GLS, ALT). This response pattern suggests a cellular stress response that does not achieve homeostasis, potentially leading to intracellular ammonia accumulation and a microenvironment that contributes to TJ damage.

This metabolic pressure may further disrupt TJ protein synthesis and trafficking by affecting intracellular ATP availability or inducing abnormal protein modifications (37), potentially contributing to the ineffective compensation phenotype. The partial normalization of nitrogen metabolism observed after ZO-1 agonist treatment may be a consequence of improved barrier integrity and a more stable intracellular environment, or it may reflect enhanced cellular stress tolerance secondary to improved structural integrity.

When compounded by the concurrent downregulation of the ECM-FA support system, this intracellular metabolic pressure creates a cellular environment in which TJ transcripts are abundantly produced but their protein products cannot be correctly folded, trafficked, or anchored at the apical membrane. The convergence of metabolic toxicity within the cell and structural anchoring failure at the basal side offers a plausible upstream explanation for the ineffective transcriptional compensation observed in this study.

### 4.5 Species-specificity of ammonia nitrogen stress response in *L. longirostris*

Although *L. longirostris* shares the family Bagridae with *P. fulvidraco*, both are economically valuable scaleless aquaculture species native to China that are susceptible to ammonia nitrogen stress (38). However, their intestinal TJ genes exhibit fundamentally divergent stress response patterns.

Under ammonia nitrogen stress, *P. fulvidraco* follows a pattern: downregulation of core barrier-forming TJ genes (*zo-1*, *occludin-1*,*claudin-1*) and specific upregulation of the pore-forming gene (*claudin-2*) (13). This transcriptional pattern is consistent with the TJ degeneration and increased barrier permeability observed at the ultrastructural level.

In *L. longirostris*, we observed a different pattern. Under ammonia nitrogen stress, TJ-related genes exhibited a non-discriminatory upregulation, affecting both barrier-forming (*zo-1*, *occludin-1*,*claudin-1*) and pore-forming genes (*claudin-2*), as well as assembly regulators (*rab13*, *actr2*). despite this transcriptional response, the functional outcome was fragmented TJ structure, reduced TEER, and disrupted protein localization (the pattern we term “ineffective compensation”).This contrasts with the response pattern of *P. fulvidraco*, highlighting species-specific adaptations in stress regulation.

### 4.6 Environmental implications, limitations, and future perspectives

The molecular decoupling between mRNA abundance and protein function observed in this study provides a perspective on how environmental stressors may compromise mucosal immunity. This phenomenon suggests that traditional biomarkers based solely on gene expression may not fully reflect functional barrier status, supporting the value of integrated assessments that combine transcriptional, proteomic, and functional readouts.

From a practical standpoint, the protective effects of the ZO-1 agonist suggest a potential strategy for enhancing resilience against chronic ammonia exposure. These findings indicate that ZO-1-targeted molecules merit further investigation as candidates for functional feed additives.

Several limitations should be acknowledged. First, the specificity and mode of action of the ZO-1 agonist (HY-N6612B) have not been directly validated in fish. Although the agonist induced negligible transcriptional changes and its protective effect was reproducible in the *ex vivo* explant model, direct evidence of target engagement in this species is lacking. In particular, the specific receptor or signaling pathway through which it acts has not been identified. Second, the “ineffective transcriptional compensation” phenotype is currently supported by mRNA-level, ultrastructural, and functional data, but has not been verified by direct quantitative protein analyses. Western blotting, protein turnover assays, and translation efficiency measurements are needed to determine whether the mRNA-protein disconnect arises from impaired translation, increased protein degradation, or defective assembly. Third, the specific post-translational modifications on ZO-1 that may mediate its delocalization under ammonia stress remain unidentified. Fourth, the upstream signaling sensors that couple ammonia-induced metabolic stress to TJ protein assembly failure are still unknown.

Future studies incorporating quantitative proteomics and targeted protein biochemical assays will be necessary to address these gaps and to refine the mechanistic framework proposed here.

## 5 Conclusion

This study shows that ammonia nitrogen stress is associated with intestinal barrier failure in *L. longirostris*, accompanied by a pattern of ineffective transcriptional compensation. Under ammonia stress, a paradoxical surge in tight junction transcripts fails to achieve functional protein assembly and correct membrane localization. Our data suggest that preservation of ZO-1-associated junctional structure is associated with partial restoration of barrier integrity and partial repair of the basal ECM-FA support system.

## Data Availability

The RNA-seq data is available in the Genome Sequence Archive (GSA) in the National Genomics Data Center, China National Center for Bioinformation/Beijing Institute of Genomics via accession number CRA037958. The title is “Intestinal transcriptome data of Leiocassis longirostris under ammonia nitrogen stress”. The project number is “PRJCA055129”, and the release date is April 1, 2026. The website link is https://ngdc.cncb.ac.cn/gsa/search?searchTerm=CRA037958.
