# Supplementary material for: Pharmacological ZO-1 agonist treatment attenuates ammonia nitrogen stress-induced mucosal inflammation and intestinal barrier failure in Leiocassis longirostris
Source: Front Immunol. 2026 Jun 8;17:1842403. doi: 10.3389/fimmu.2026.1842403 (PMC13284113; doi:10.3389/fimmu.2026.1842403)
Supplement: Supplementary file 1 [file SupplementaryFile1.docx]

**Table S1. The 24h median lethal concentration of ammonia nitrogen to *Leiocassis longirostris (in vivo)***

| ammonia nitrogen  concentration (mg/L) | The number of test fish | The number of deaths | Mortality rate (%) | 24h LC50  (mg/L) |
| --- | --- | --- | --- | --- |
| 0 | 30 | 0 | 0 | 26.35 |
| 10 | 30 | 2 | 6.67 |  |
| 20 | 30 | 7 | 23.33 |  |
| 30 | 30 | 15 | 50.00 |  |
| 40 | 30 | 23 | 76.67 |  |
| 50 | 30 | 30 | 100 |  |

**Table S2. The effects of different concentrations of ZO-1 agonists on the test fish within 24 h (*in vivo*)**

| Agonist concentration (mM) | The number of test fish | Mortality rate (%) | Abnormal rate of body surface mucus/behavior (%) | *ZO-1*  expression | *Occludin*  expression |
| --- | --- | --- | --- | --- | --- |
| 0 | 10 | 0 | 0 | 1.00 ± 0.08 | 1.00 ± 0.06 |
| 2.5 | 10 | 0 | 0 | 1.62 ± 0.12* | 1.58 ± 0.09* |
| 5 | 10 | 0 | 0 | 2.85 ± 0.15* | 2.79 ± 0.11* |
| 10 | 10 | 0 | 20 | 2.36 ± 0.10* | 2.89 ± 0.08* |

**P* < 0.05

**Table S3. Effects of different ammonia nitrogen concentrations on intestinal explants after 6 h of stress (*ex vivo*)**

| TAN Concentration (Relative to in vivo) | TAN (mg/L) | TEER (% of Initial) Mean ± SD | LDH Release Rate (%) Mean ± SD |
| --- | --- | --- | --- |
| 0 (Control) | 0.0 | 88.90 ± 3.37 | 8.36 ± 1.25 |
| 1/30 in vivo | 0.43 | 78.46 ± 2.83* | 10.43 ± 0.71 |
| 1/20 in vivo | 0.65 | 59.83 ± 5.09* | 13.93 ±1.62* |
| 1/10 in vivo | 1.30 | 25.00 ± 5.01* | 29.83 ± 4.02* |

The ammonia nitrogen (TAN) concentration selected for the i*n vivo* experiment was 13 mg L⁻¹ (equivalent to 1/2 24h LC50). **P* < 0.05

| Gene name | primer sequence (5′-3′) | Reaction Efficiency（%） | GenBank accession number |
| --- | --- | --- | --- |
| *glud* | F: CGTCCCTGTTCCAGTGTGAT | 98.2 | XM_060872857.1 |
|  | R: TCTTCAGGATCCCCCGGAC |  |  |
| *glul* | F: GCGCATATCTGACCCTCCTG | 98.4 | XM_060867082.1 |
|  | R: ACATCAGCGCGAGTACATCA |  |  |
| *slc1a5* | F: ATCCGTTAGGAGGTCACCGA | 98.8 | XM_060888866.1 |
|  | R: AGCATTCATAGAGAAGGCTGGA |  |  |
| *aqp1* | F: GGTGAAGGTGTCTCTGGCAT | 97.2 | [XM_060875380.1](https://www.ncbi.nlm.nih.gov/entrez/viewer.fcgi?db=nucleotide&id=2618783660) |
|  | R: TGTGGTGCACTGATGCTGT |  |  |
| *zo-1* | F: GCGAACTCTCTGAACAGCCT | 96.3 | XM_047810112.1 |
|  | R: TGTGTGTGTGCAGGAGGTTT |  |  |
| *occludin* | F: CGAGCGAGAGACTACGACAC | 95.8 | XM_027141818.2 |
|  | R: TCCAGGAATTGTGGGCTTCC |  |  |
| *claudin-1* | F: ACGCTAACAACGGCTCAGA | 97.2 | XM_047815958.1 |
|  | R: CCTTACATTCAGACACCACCTT |  |  |
| *claudin-2* | F: TCAGGACGACAGACGAGGA | 98.3 | XM_047804898.1 |
|  | R: AGGCACACCCACAGGAACT |  |  |
| *rab13* | F: AGTCCAAATCTGTGTGTCTCTCCC | 98.8 | XM_060870810.1 |
|  | R: AAGATTCGTCGGCATTTCGC |  |  |
| *actr* | F: ATCACTGCGGCTGATTGAGT | 98.5 | XM_060896752.1 |
|  | R: CACTAACACCCTCACGCTCT |  |  |
| *col1a1* | F: GCTCTCTACAGGGTGCTGC | 99.1 | XM_060897661.1 |
|  | R: GTCCACGACCACCAATAGGA |  |  |
| *thbs* | F: TTTTACGTGTGCCTTGTGCC | 99.3 | XM_060866432.1 |
|  | R: TGGAAGCACTAAAATGGACAAACA |  |  |
| *fn1* | F: AGGAGAGTTCAAGTGCGAGC | 96.8 | XM_060876754.1 |
|  | R: TAAGGCCTACCAGTTTGCGT |  |  |
| *itga* | F:GCTTTGGGACAAAAGTCCGTC | 98.3 | XM_060895416.1 |
|  | R: ATGTGTCTCACTCACGGCAG |  |  |
| *itgb* | F: GTACATTCGGCTGGTGAGGG | 96.9 | XM_060867595.1 |
|  | R: ACTTGGAGGCATTCTGCTGT |  |  |
| *rhoa* | F: CTCCGACCGCTTTCCTATCC | 97.1 | XM_060879806.1 |
|  | R: GCAAGTTCCCATAGGCATTACG |  |  |
| *rock* | F: CGTTTCACTGGGACGGAGAA | 98.1 | XM_060875375.1 |
|  | R: GGCCAAGGTTTCGCCAATTT |  |  |
| *mylk2* | F: ATCATGTCCCCTGCTTGGTC | 97.9 | XM_060894081.1 |
|  | R: AGCAGGCAACAAGCTACAGA |  |  |
| *β-actin* | F:GGCATCCATGAGACCACCTT | 99.5 | JN833583.1 |
|  | R: CCACCTGTACCAAGCCACAA |  |  |

**Table S4. Primer sequences in this study**


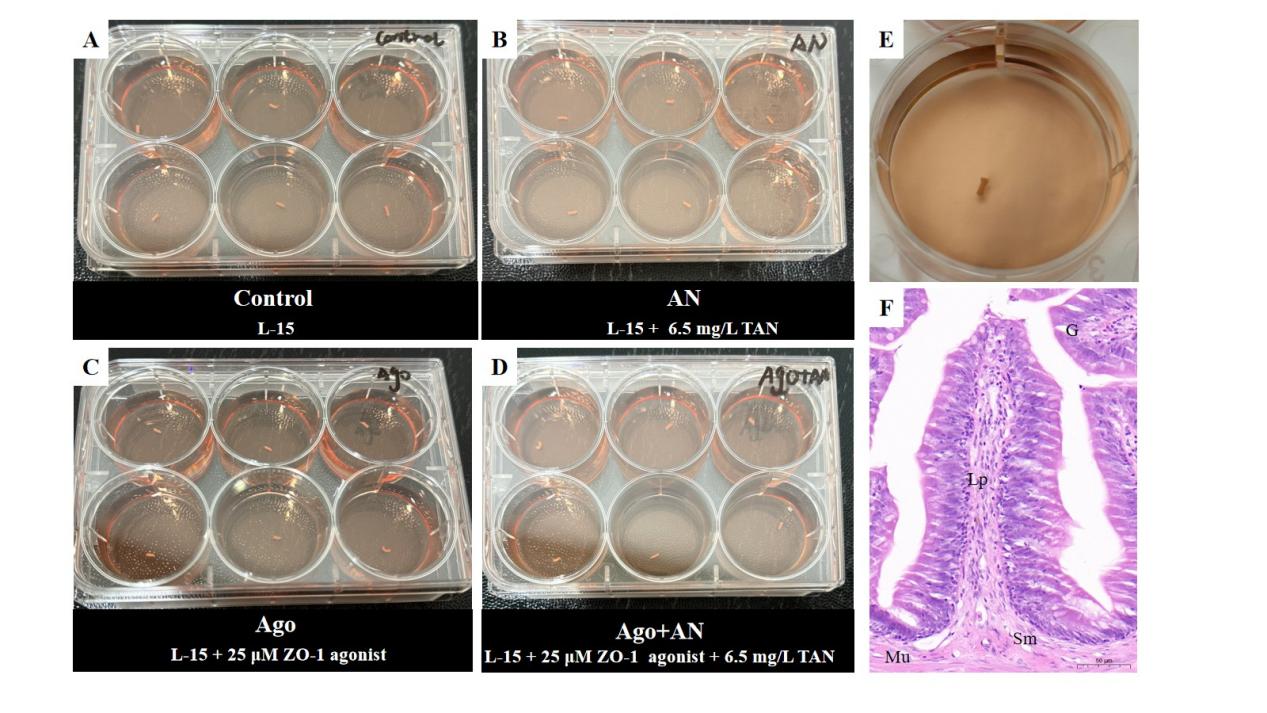


**Figure S1. *Ex vivo* experiments of intestinal explants**

**(A-D)** The experimental treatments for the intestinal explants in the Control, AN, Ago, and Ago + AN groups respectively. The duration of the treatment was 6 h. **(E)** Control group intestinal explants. After 6 h of *ex vivo* culture, there were no significant changes in morphology and color, and no obvious mucus accumulation occurred. Therefore, the intestinal explants could survive well in the L-15 medium for a short period of time. **(F)** H&E staining of the *ex vivo* intestinal tissues in the Control group. The organizational structure is complete, with no obvious signs of edema or necrosis, indicating that the isolated tissues could remain viable during the experiment.

Mu, mucosa; Sm, submucosa; Lp, lamina propria; G, mucous cell.


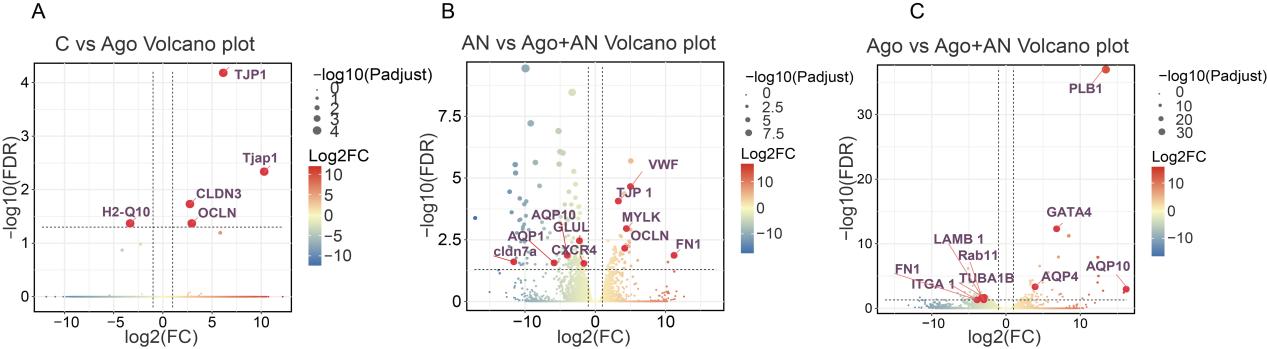


**Figure S2. Volcano plots of DEGs**

**(A-C)** Volcano plots of DEGs among Control vs. Ago, AN vs. Ago+AN, Ago vs. Ago+AN, respectively. Orange dots represent up-regulated DEGs, blue dots represent down-regulated DEGs, and yellow dots represent non-differentially expressed genes. Selected key gene names are labeled.


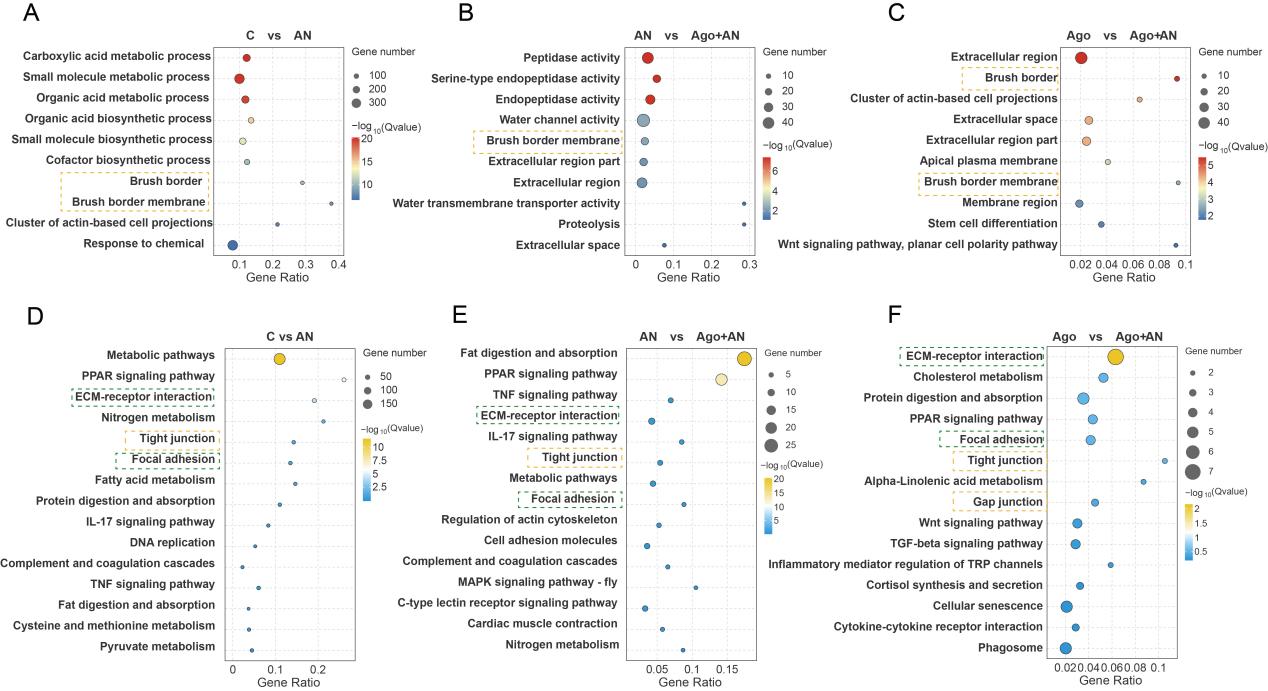


**Figure S3. GO and KEGG enrichment analysis**

**(A-C)** GO enrichment analysis of DEGs among Control vs. AN, AN vs. Ago+AN, Ago vs. Ago+AN, respectively. **(D-F)** KEGG enrichment analysis of DEGs among Control vs. AN, AN vs. Ago+AN, Ago vs. Ago+AN, respectively.

The vertical axis lists the GO terms/pathway, and the horizontal axis Rich factor represents the ratio of sample number/background number. Dot size corresponds to the number of genes in the term, and color represents the adjusted p-value (-log₁₀).
